# Hospital-acquired acute kidney injury in very elderly men: clinical characteristics and short-term outcomes

**DOI:** 10.1007/s40520-019-01196-5

**Published:** 2019-06-10

**Authors:** Qinglin Li, Meng Zhao, Feihu Zhou

**Affiliations:** 1 Department of Critical Care Medicine, the First Medical Center, Chinese PLA General Hospital, 28 Fuxing Road, Beijing, 100853 China; 2 Department of Clinical Data Repository, Chinese PLA General Hospital, Beijing, 100853 China

**Keywords:** Acute kidney injury, Very elderly, Incidence, Prognosis, Risk factors

## Abstract

**Objectives:**

We explored the risk factors for, and the clinical characteristics of, acute kidney injury (AKI), and the causes of death 28 days after such injury, in very elderly men.

**Methods:**

This was a retrospective cohort study using data from the Geriatric Department of the Chinese PLA General Hospital. A total of 3464 elderly patients (≥ 75 years) were enrolled from January 2007 to December 2015. All patients were followed for 28 days or until death after AKI.

**Results:**

In total, 668 patients (39.0%) developed AKI, and 623 men were included for the final analysis. The median age was 87 years. The 28-day mortality rate was 25.7%. The AKI etiologies were infections (39.6%), hypovolemia (23.8%), cardiovascular events (15.9%), nephrotoxicity (12.0%), and surgery (7.1%). Multiple organ dysfunction syndrome (46.4%) and pulmonary infection (22.5%) were the principal causes of death. Multivariate analysis revealed that time for AKI to develop (HR = 0.865; 95% CI 0.799–0.937; *P *< 0.001), low mean arterial pressure (HR = 0.970; 95% CI 0.958–0.981; *P *< 0.001), low serum prealbumin (HR = 0.924; 95% CI 0.894–0.955; *P *< 0.001) level, oliguria (HR = 2.261; 95% CI 1.424–3.590; *P *= 0.001), mechanical ventilation (HR = 1.492; 95% CI 1.047–2.124; *P *= 0.027), blood urea nitrogen (HR = 1.037; 95% CI 1.025–1.049; *P *< 0.001) level, magnesium (HR = 2.512; 95% CI 1.243–5.076; *P *= 0.010) level, and more severe AKI stages (stage 2: HR = 3.709; 95% CI 1.926–7.141; *P *< 0.001 and stage 3: HR = 5.660; 95% CI 2.990–10.717; *P *< 0.001) were independent risk factors for 28-day mortality.

**Conclusions:**

The incidence of AKI increases significantly as age advanced. Identification of risk factors might lead to more intensive monitoring and early prevention, and might improve AKI patients’ outcomes in the very elderly.

## Introduction

Acute kidney injury (AKI), previously termed as acute renal failure (ARF), has attracted increasing attention in recent decades because of its rising incidence and its adverse effect on patients. In the elderly, age-dependent structural and functional changes make an old kidney more sensitive to AKI [1, 2]. An analysis showed that the incidence of AKI increases stepwise from 24.9 episodes (75–79 years) to 34.2 episodes (80–84 years) to 46.9 episodes (85 years and older) per 1000 patient-years, respectively [3]. Despite this, few studies have specifically focused on elderly patients with AKI.

AKI has been recognized as a surrogate marker of the severity of illness. This complex clinical disorder is closely associated with both short- and long-term mortality, the length of hospital stay, as well as a predictor of chronic kidney disease (CKD) [4–7]. However, focusing on the long-term outcomes of geriatric AKI individuals may often be difficult because they have significantly poor prognosis after hospital discharge, and survivors are often left with multiple chronic conditions [2, 4]. The long-term survival rate might be reduced significantly; so short-term consequences, such as in-hospital mortality, may be more important and practical.

AKI has become a huge medical burden in China [8]. The clinical features of elderly AKI patients in China are poorly known, and very few studies on risk factors predicting short-term (28-day) outcomes and causes of death post-AKI have appeared. In the present study, we described the risk factors for mortality among AKI clinical characteristics, and the causes of death by 28 days post-AKI in very elderly patients.

### Patients and methods

This was a retrospective cohort study performed in the Geriatric Department of the Chinese PLA General Hospital. We collected data on very elderly patients (≥ 75 years of age) treated from January 1, 2007, to December 31, 2015. All patients who developed AKI were enrolled. All patients who developed AKI were enrolled. Patients were divided into survivor and non-survivor groups by their outcomes within 28 days after AKI. We sought clinical risk factors for death and explored their effects.

AKI was diagnosed by reference (exclusively) to SCr level, thus by an SCr increase ≥ 0.3 mg/dl (≥ 26.5 µmol/L) within 48 h, or an increase to ≥ 1.5-fold the baseline value, known or presumed to have developed within the prior 7 days [9]. AKI severity was scored by reference to the difference between SCr baseline and peak values, using the KDIGO staging criteria: Stage 1, an increase ≥ 26.5 µmol/L, or a rise to ≥ 1.5- to 1.9-fold the baseline value; Stage 2, a rise to ≥ 2.0- to 2.9-fold the baseline value; Stage 3, a rise to ≥ 3.0-fold the baseline value, or to ≥ 353.6 μmol/L, or initiation of renal replacement therapy. We noted age, AKI etiology, gender, body mass index (BMI), co-morbidities (hypertension, chronic obstructive pulmonary disease [COPD], coronary disease, diabetes mellitus), recently performed surgical operations, need for dialysis, need for MV, and cause of death. MV was defined as ventilation for at least 24 h prior to AKI development. Oliguria was defined as urinary output < 400 ml/24 h. Urine output, mean arterial pressure (MAP), and the levels of SCr, blood urea nitrogen (BUN), uric acid, hemoglobin, serum albumin, serum prealbumin, serum magnesium, serum calcium and serum phosphate were also noted at the time of AKI diagnosis. The baseline SCr level was the most recent stable measure taken in the 1–3 months prior to admission with AKI [10, 11]. Estimated glomerular filtration rates (eGFR) were calculate by the Chronic Kidney Disease Epidemiology Collaboration (CKD-EPI) [12].

We excluded patients younger than 75 years, those with previously diagnosed CKD [13], those who stayed in the hospital for less than 48 h, those who had only one SCr or no SCr examination, those who had missing or incomplete medical history and those who had early death within 48 h after admission. We also excluded females, because we treated fewer females than males.

### Statistical analysis

Continuous variables were presented as means ± standard deviations (SDs), or the median (25–75% interquartile range), depending on the variable distribution. Discrete variables are presented as counts or percentages. Statistical analyses were performed using SPSS version 17.0 for Windows (SPSS Inc., Chicago, IL, USA). Between-group comparisons were made using Student’s *t*-tests or Mann–Whitney U-test. Correlations between potential risk factors and death were assessed using Pearson’s Chi-squared or Fisher’s exact tests. Survival curves were estimated by the Kaplan–Meier product-limit method and compared by the Mantel (log-rank) test. Prognostic factors of survival were identified by the use of the Cox proportional hazards regression model. A *P* value < 0.05 was considered to reflect statistical significance.

## Results

### Baseline data of the enrolled patients

**Fig. 1:**
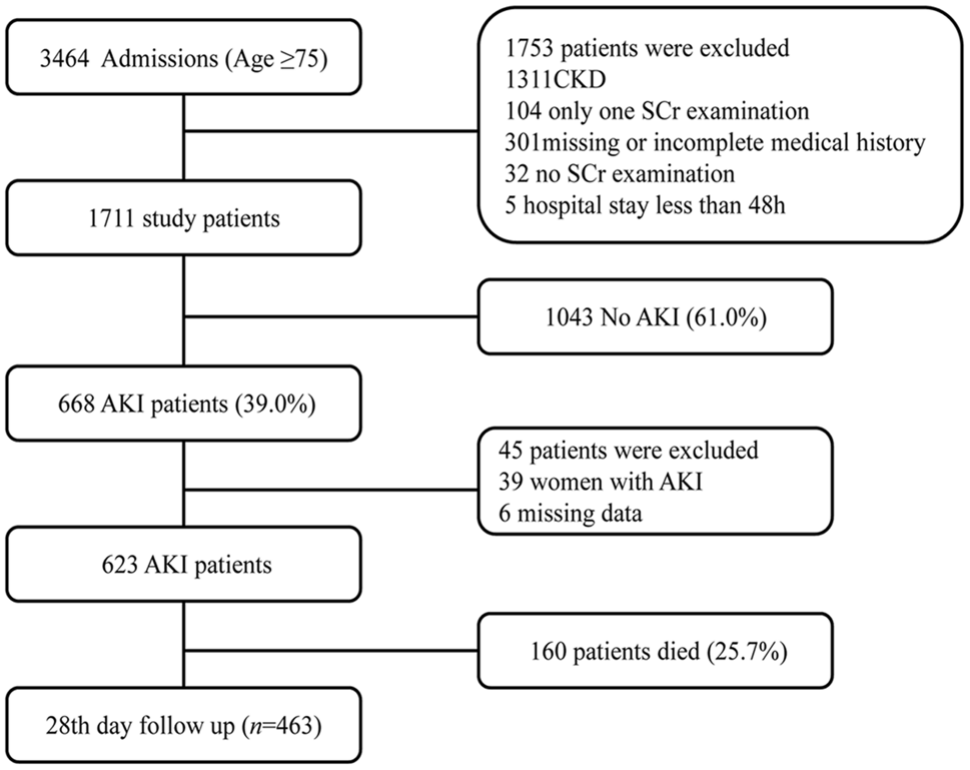
Flow chart of patients included and excluded in the study

Among 1711 study patients, 668 (39.0%) developed AKI. Only 45 patients were excluded, resulting in 623 AKI patients suitable for analysis. The study flow chart is presented as Fig. 1. Table 1 shows the age-related incidence of AKI in the elderly patients.

**Table 1:** Age-related incidence of AKI in the elderly patients

Age (years)	AKI patients	Total inpatients	Incidence (%)
75–79	76	541	14.0
80–84	135	467	28.9
85–89	233	438	53.2
90	224	265	84.5
Total	668	1711	39.0

*AKI* Acute kidney injury

### Demographic characteristics of AKI patients

Baseline characteristics of 623 AKI patients are shown in Table 2. The median age of the cohort was 87 years. The median baseline SCr level was 74.0 μmol/L, the baseline eGFR was 78.4 ml/min/1.73^2^. Using the KDIGO criteria, 294 patients (47.2%) had stage 1 AKI, 157 (25.2%) had stage 2, and 172 (27.6%) had stage 3. In all, 160 (25.7%) died within 28 days. The predominant comorbidities were coronary disease 487 (78.2%), hypertension 460 (73.8%), COPD 438 (70.3%), and diabetes mellitus 223 (35.8%). Overall, 231 (37.1%) required MV, 35 (5.6%) had oliguria, and 9 (1.4%) of the stage 3 patients acquired acute dialysis.

**Table 2:** Comparisons of the clinical characteristics between elderly AKI survivors and non-survivors

Characteristic	AKI patients (*n* = 623)	Non-survivors (*n* = 160)	Survivors (*n* = 463)	*P*
Age (years)	87 (84–91)	87 (83–90)	87 (84–91)	0.235
BMI (kg/m^2^)	23.0 ± 3.1	22.4 ± 2.8	23.2 ± 3.2	0.004
Comorbidity				
Coronary disease	487 (78.2)	126 (78.8)	361 (78.0)	0.837
Hypertension	460 (73.8)	115 (71.9)	345 (74.5)	0.499
COPD	438 (70.3)	109 (68.1)	329 (71.1)	0.538
Diabetes	223 (35.8)	58 (36.3)	165 (35.6)	0.833
Baseline SCr (μmol/L)	74.0 (62.0–84.0)	66.0 (54.0–75.8)	78.0 (65.0–86.0)	<0.001
Baseline eGFR (ml/min/1.73^2^)	78.4 (71.3–84.9)	83.1 (77.0–90.7)	77.0 (69.7–83.0)	<0.001
Etiology of AKI				
Infections	247 (39.6)	81 (50.6)	166 (35.9)	0.001
Hypovolemia	148 (23.8)	41 (25.6)	107(23.1)	0.411
Cardiovascular events	99 (15.9)	20 (12.5)	79 (17.1)	0.167
Nephrotoxicity	75 (12.0)	8 (5.0)	67 (14.5)	0.003
Surgery	44 (7.1)	9 (5.6)	35 (7.6)	0.348
Others	10 (1.6)	1 (0.6)	9 (1.9)	0.321
Time for AKI to develop (days)	2.0 (2.0–7.0)	2.0 (1.0–4.0)	4.0 (2.0–7.0)	<0.001
Parameter at the time of AKI diagnosis				
MAP (mmHg)	78 ± 14	70 ± 13	81 ± 14	<0.001
Oliguria	35 (5.6)	23 (14.4)	12 (2.6)	<0.001
Dialysis	9 (1.4)	2 (1.3)	7 (1.5)	0.714
MV	231 (37.1)	111 (69.4)	120 (25.9)	<0.001
Laboratory results at the time of AKI diagnosis				
SCr (μmol/L)	132.0 (118.2–147.0)	141.5 (125.1–162.0)	128.9 (117.2–142.0)	<0.001
Peak SCr (μmol/L)	144.0 (124.8–204.0)	218.5 (159.1–318.8)	136.9 (121.7–166.7)	<0.001
BUN (mmol/L)	12.8 (8.9–21.3)	23.6 (15.9–36.3)	11.0 (8.2–16.6)	<0.001
Uric acid (μmol/L)	367.0 (293.7–471.0)	424.4 (329.8–556.4)	355.0 (281.0–440.2)	<0.001
Prealbumin (g/L)	181.0 (139.0–234.0)	139 (108–176)	200 (154–252)	<0.001
Albumin (g/L)	34.3 ± 5.5	30.8 ± 5.1	36.0 ± 5.1	<0.001
Magnesium (mmol/L)	0.9 (0.8–1.0)	1.0 (0.8–1.1)	0.9 (0.8–1.0)	0.009
Calcium (mmol/L)	2.2 (2.1–2.3)	2.2 (2.0–2.3)	2.2 (2.1–2.4)	0.069
Phosphate (mmol/L)	1.2 (1.0–1.4)	1.3 (1.0–1.6)	1.2 (1.0–1.4)	<0.001
Hemoglobin (g/L)	112 ± 23	102 ± 23	116 ± 21	<0.001
AKI stage				<0.001
1	294 (47.2)	12 (7.5)	282 (60.9)	
2	157 (25.2)	47 (29.4)	110 (23.8)	
3	172 (27.6)	101 (63.1)	71 (15.3)	

Values are *n* (%), mean ± SD or median (inter-quartile range)

*AKI* Acute kidney injury, *BMI* body mass index, *COPD* chronic obstructive pulmonary disease, *MAP* mean arterial pressure, *1* *mmHg* 133 kPa, *MV* mechanical ventilation, *SCr* serum creatinine, *BUN* blood urea nitrogen

### Etiology of AKI in geriatric patients

**Fig. 2:**
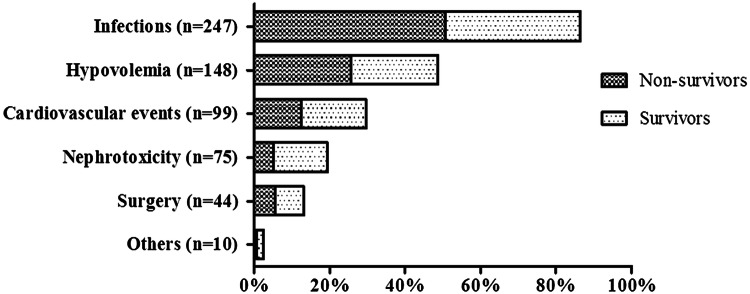
Etiology of acute kidney injury

The most frequent causes of AKI in geriatric patients were infections 247 (39.6%), hypovolemia 148 (23.8%; volume depletion, or hypotension), cardiovascular events 99 (15.9%; acute coronary syndrome or acute heart failure), nephrotoxicity 75 (12.0%), and surgery 44 (7.1%) (Fig. 2).

### Clinical characteristics and outcome

As shown in Table 2, no significant difference was evident between non-survivors and survivors in terms of age (*P *= 0.235) and comorbidities (*P *= 0.837, 0.499, 0.538, and 0.833, respectively). Mean BMI (22.4 ± 2.8 kg/m^2^ vs. 23.2 ± 3.2 kg/m^2^, *P *= 0.004), baseline SCr value (66.0 μmol/L vs. 78.0 μmol/L, *P *< 0.001) and baseline eGFR (83.1 ml/min/1.73^2^ vs. 77.0 ml/min/1.73^2^, *P *< 0.001) differed significantly between the two groups.

As shown in Table 2, non-survivors were more likely to have had infections and to have required MV upon AKI diagnosis than were survivors (50.6% vs. 35.9%, *P *=0.001; 69.4% vs. 25.9%, *P *<0.001). Of the 160 patients non-survived, AKI diagnosis occurred 2.0 days (1.0–4.0 days) compared to 4.0 days (2.0–7.0 days) with patients survived (*P *< 0.001). Low MAP (70 ± 13 mmHg vs. 81 ± 14 mmHg, *P *< 0.001), oliguria (14.4% vs. 2.6%, *P* < 0.001), anemia (102 ± 23 g/L vs. 116 ± 21 g/L, *P *< 0.001), a lower prealbumin level (139 g/L vs. 200 g/L, *P *< 0.001), and hypoalbuminemia (30.8 ± 5.1 g/L vs. 36.0 ± 5.1 g/L, *P *< 0.001), higher uric acid (424.4 μmol/L vs. 355.0 μmo/L, *P *< 0.001) level, magnesium (1.0 mmol/L vs. 0.9 mmo/L, *P *= 0.009) level and phosphate (1.3 mmol/L vs. 1.2 mmo/L, *P *< 0.001) level were more frequent among non-survivors. As expected, the non-survivors had higher SCr, BUN, and peak SCr levels at the time of AKI diagnosis (all *P *< 0.001).

**Fig. 3:**
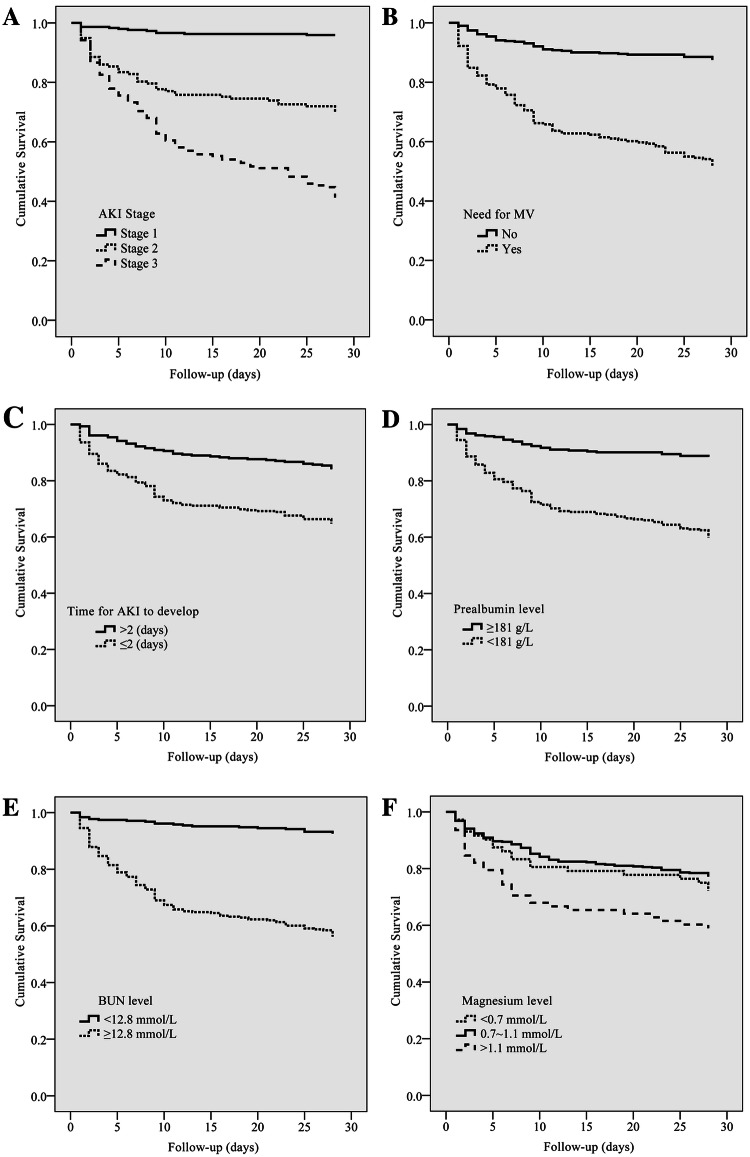
**a** Kaplan–Meier survival curves for 28-day mortality by KDIGO Stages (log-rank test: *P *< 0.001). **b** Kaplan–Meier survival curves for 28-day mortality in patients with and without MV (log-rank test: *P *< 0.001). **c** Kaplan–Meier survival curves for 28-day mortality according to time for AKI to develop, using 2 days as the cutoff point (log-rank test: *P *< 0.001). **d** Kaplan–Meier survival curves for 28-day mortality according to prealbumin level, using the median values as the cutoff point (181 g/L) (log-rank test: *P *< 0.001). **e** Kaplan–Meier survival curves for 28-day mortality according to BUN level, using the median values as the cutoff point (12.8 mmol/L) (log-rank test: *P *< 0.001). **f** Kaplan–Meier survival curves for 28-day mortality according to magnesium level, using the normal values as the cutoff point (0.7– 1.1 mmol/L as normal values) (log-rank test: *P *= 0.001)

Table 2 also shows the relationship between the AKI stage and short-term outcome; AKI severity was associated with a significantly higher 28-day mortality (7.5% for stage 1 patients, 29.4% for stage 2, and 63.1% for stage 3). Unsurprisingly, outcomes worsened with more advanced AKI stage (*P *< 0.001 for the three stages, respectively) (Fig. 3a).

### Risk factors for 28-day mortality in geriatric AKI patients

Multivariate analysis by the Cox model revealed that time for AKI to develop (HR = 0.865; 95% CI 0.799–0.937; *P *< 0.001), low MAP (HR = 0.970; 95% CI 0.958–0.981; *P *< 0.001), low prealbumin level (HR = 0.924; 95% CI 0.894–0.955; *P *< 0.001), oliguria (HR = 2.261; 95% CI 1.424–3.590; *P *= 0.001), requirement for MV (HR = 1.492; 95% CI 1.047–2.124; *P *= 0.027), BUN level (HR = 1.037; 95% CI 1.025–1.049; *P *< 0.001), magnesium level (HR = 2.512; 95% CI 1.243–5.076; *P *= 0.010) and more severe AKI stage (stage 2 compared 1: HR = 3.709; 95% CI 1.926–7.141; *P *< 0.001; stage 3 compared 1: HR = 5.660; 95% CI 2.990–10.717; *P *< 0.001) were independent risk factors for mortality in those who died within 28 days after AKI diagnosis (Table 3).

**Table 3:** Multivariate Cox proportional hazard model analysis of risk factors for 28-day mortality

Risk factor	HR	95% CI	*P*
Time for AKI to develop	0.865	0.799–0.937	<0.001
MAP	0.970	0.958–0.981	<0.001
Prealbumin level	0.924	0.894–0.955	<0.001
Oliguria	2.261	1.424–3.590	0.001
MV	1.492	1.047–2.124	0.027
BUN level	1.037	1.025–1.049	<0.001
Magnesium level	2.512	1.243–5.076	0.010
AKI stage			<0.001
Stage 1	Reference	Reference	
Stage 2	3.709	1.926–7.141	<0.001
Stage 3	5.660	2.990–10.717	<0.001

*HR* Hazard ratio, *CI* confidence interval, *MAP* mean arterial pressure, *1* *mmHg* 0.133 kPa, *MV* mechanical ventilation, *BUN* blood urea nitrogen, *AKI* acute kidney injury

According to the Kaplan–Meier plot, the survival curve of patients with no MV had a significantly better survival than patients with MV (Fig. 3b). Patients diagnosis AKI > 2 days had better survival than diagnosis AKI in 2 days (Fig. 3c). Patients with prealbumin level ≥ 181 g/L had better survival than patients with lower level (Fig. 3d). Patients with BUN level ≥ 12.8 mmol/L were significantly worse than the patients with lower BUN level (Fig. 3e). Patients with hypomagnesemia had better survival than patients with hypermagnesemia (Fig. 3f).

### Cause of death

A total of 160 of the 623 AKI patients (25.7%) died within 28 days of AKI diagnosis. Multiple organ dysfunction syndrome (MODS, 79 cases, 49.4%) and pulmonary infection (36, 22.5%) were the principal causes of death. Other causes included respiratory failure (18, 11.3%), cardiovascular events (17 cases, 10.6%), hemorrhagic shock (4, 2.5%), ARF (1, 0.6%) and others (5, 3.1%).

## Discussion

We retrospectively studied very elderly patients with AKI over 28 days of follow-up to understand their characteristics, AKI etiologies, and hospital outcomes. To the best of our knowledge, no previous study has evaluated short-term mortality after AKI in the very elderly patients, especially standardized complete KDIGO criteria.

In the very elderly, the development of clinically relevant AKI is associated with age-dependent changes, which are independent risk factors for AKI. Such changes render older patients more prone to medication-associated toxicities and ischemic injury. Furthermore, elderly individuals are very vulnerable to co-existing illness and are more likely to require surgery. Often, multi-drug combination therapy is required; this is a prime trigger of AKI. Previous research reported the incidence of AKI among very elderly patients as 23–40%, with most patients classified as stage 1 (RIFLE or AKIN criteria) [14–16]. We found that 39.0% of geriatric patients had AKI, in agreement with the literature [11].

As in other works [17, 18], we found that infections (39.6%), hypovolemia (23.8%), cardiovascular events (15.9%), nephrotoxicity (12.0%) were the principal causes of AKI. Infections were the prime cause, almost always preceded by pneumonia. Elderly patients are particularly prone to pneumonia because of an impaired gag reflex, decreased mucociliary function, waning immunity, and increasing cardiopulmonary dysfunction [19]. In the very elderly, coexisting illnesses play major roles in triggering pneumonia, and they influence disease severity [20].

The in-hospital mortality of our elderly AKI patients (25.7%) was slightly higher than in previously published studies (16–20%) [11, 21], but lower than the rate of 33–40% reported in previous reports on patients older than 75 years [17, 22]. The wide variation in the mortality of elderly AKI stems from the population being studied, etiology to AKI, the ICU settings, the definition of AKI used and whether CKD is included during the studies. In our cohort, of the 172 stage 3 AKI patients, only nine patients required dialysis during follow-up, and 2 died within 28 days. The most common causes of death were pneumonia-associated MODS (49.4%) and pulmonary infections (22.5%). Other less frequent causes of death were respiratory failure (11.3%), cardiovascular events (10.6%), hemorrhagic shock (2.5%), ARF (0.6%) and other (3.1%). Immunity often wanes in the elderly. High comorbidity levels, age-related changes in organ function, and diminishing organ reserves render the elderly more vulnerable to pneumonia, causing AKI patients to progress to MODS [23]. In the present study, the second leading cause of death was pulmonary infection (22.5%), emphasizing that pneumonia caused either AKI or the lethal MODS.

Many studies have demonstrated the importance of age and male gender as risk factors for death in AKI [8, 14, 21]. In the present study, the median age was 87 years, and there was no significant difference of ages among survivors and non-survivors. This was probably because this is a geriatric hospital; almost health objects are male gender.

In the present study, there were some significant factors associated with death, such as lower MAP, oliguria and need for MV. The presentation of lower MAP in non-survivors is interesting. We suggest that this manifestation might result from the causes of AKI. In our study, 25.6% of the non-survivors were related to hypovolemia and it is expected that those who died with AKI might exhibit hypotension more frequent than those who survived. This suggests that the occurrence of hypotension, oliguria and MV is associated with a higher risk of death.

Malnutrition is a common problem in elderly patients. Several studies on the association between mortality and nutritional status have found that preexisting malnutrition is associated with poor outcomes in AKI patients [24, 25]. In our study, the prealbumin levels of non-survivors were significantly lower than those of survivors; prealbumin levels of 181 g/L were strongly associated with a higher risk of 28-day mortality.

Previous research suggests that BUN is predictive for short- and long-term mortality independent of SCr level [26]. In patients with severe AKI who required dialysis, predialysis BUN level is predictive of 60-day mortality [27]. We found BUN level (≥ 12.8 mmol/L) to be independent predictors of short-term mortality. SCr was not associated with 28-day mortality after adjustment for other covariates.

The effects of dysmagnesemia on mortality remain controversial. Previous studies reported nearly twice mortality rates in hypomagnesemic compared to normomagnesemic patients [28–30]. However, another research reported no differences in mortality between hypomagnesemic and normomagnesemic patients [31]. In the present study, we did not find any significant effect of hypomagnesemia on patient mortality. But we find that hypermagnesemia was strongly associated with 28-day mortality.

Our study had some potential weaknesses. First, it was a single-center retrospective work, and the results may not be immediately applicable to other hospitalized patients. Second, we did not use the urine output criteria because these data were incomplete. Third, there were fewer female treated than males. So, biased results may be unavoidable. Fourth, the study excluded patients with CKD. As CKD is a potent risk factor for AKI, excluding patients with CKD in this analysis may have potentially unestimated the rate of AKI in the very elderly. Fifth, the follow-up duration was short. We did not study patients with less severe AKI (those who survived for 28 days or longer). We also did not explore the effects of AKI on kidney outcome.

## Conclusion

There are important risk factors for death among very elderly patients with AKI that must be identified early to decrease mortality. Our findings support the view that the incidence of AKI increases significantly as age advanced. 28-day mortality is less severe than expected. Time for AKI to develop, low MAP, low prealbumin level, oliguria, requirement for MV, BUN level, magnesium level, more severe AKI stages are risk factors associated with short-term outcomes. Identification of these factors might lead to more intensive monitoring and early prevention, and might improve AKI patients’ outcomes in the very elderly.

## References

[1] Anderson S, Eldadah B, Halter JB (2011). Acute kidney injury in older adults. J Am Soc Nephrol.

[2] Coca SG (2010). Acute kidney injury in elderly persons. Am J Kidney Dis.

[3] Collins AJ, Foley RN, Herzog C (2013). US renal data system 2012 annual data report. Am J Kidney Dis.

[4] Pannu N, James M, Hemmelgarn B (2013). Association between AKI, recovery of renal function, and long-term outcomes after hospital discharge. Clin J Am Soc Nephrol.

[5] Chawla LS, Kimmel PL (2012). Acute kidney injury and chronic kidney disease: an integrated clinical syndrome. Kidney Int.

[6] Rewa O, Bagshaw SM (2014). Acute kidney injury—epidemiology, outcomes and economics. Nat Rev Nephrol.

[7] Nisula S, Kaukonen KM, Vaara ST (2013). Incidence, risk factors and 90-day mortality of patients with acute kidney injury in Finnish intensive care units: the FINNAKI study. Intensive Care Med.

[8] Yang L, Xing G, Wang L (2015). Acute kidney injury in China: a cross-sectional survey. Lancet.

[9] Eknoyan G, Lameire N, Eckardt K (2012). Kidney disease: improving global outcomes (KDIGO) acute kidney injury work group. KDIGO clinical practice guideline for acute kidney injury. Kidney Int Suppl.

[10] Hsu CY, Chertow GM, McCulloch CE (2009). Nonrecovery of kidney function and death after acute on chronic renal failure. Clin J Am Soc Nephrol.

[11] Chao CT, Tsai HB, Wu CY (2015). The severity of initial acute kidney injury at admission of geriatric patients significantly correlates with subsequent in-hospital complications. Sci Rep.

[12] Levey AS, Stevens LA, Schmid CH (2009). A new equation to estimate glomerular filtration rate. Ann Intern Med.

[13] Levin A, Stevens PE (2014). Summary of KDIGO 2012 CKD Guideline: behind the scenes, need for guidance, and a framework for moving forward. Kidney Int.

[14] Chao CT, Lin YF, Tsai HB (2013). Acute kidney injury network staging in geriatric postoperative acute kidney injury patients: shortcomings and improvements. J Am Coll Surg.

[15] Elmistekawy E, McDonald B, Hudson C (2014). Clinical impact of mild acute kidney injury after cardiac surgery. Ann Thoracic Surg.

[16] Reents W, Hilker M, Borgermann J (2014). Acute kidney injury after on-pump or off-pump coronary artery bypass grafting in elderly patients. Ann Thorac Surg.

[17] Ali T, Khan I, Simpson W (2007). Incidence and outcomes in acute kidney injury: a comprehensive population-based study. J Am Soc Nephrol.

[18] Dennen P, Douglas IS, Anderson R (2010). Acute kidney injury in the intensive care unit: an update and primer for the intensivist. Crit Care Med.

[19] Fernandez-Sabe N, Carratala J, Roson B (2003). Community-acquired pneumonia in very elderly patients: causative organisms, clinical characteristics, and outcomes. Medicine.

[20] Rello J (2008). Demographics, guidelines, and clinical experience in severe community-acquired pneumonia. Crit Care.

[21] Chao CT, Wu VC, Lai CF (2012). Advanced age affects the outcome-predictive power of RIFLE classification in geriatric patients with acute kidney injury. Kidney Int.

[22] Akposso K, Hertig A, Couprie R (2000). Acute renal failure in patients over 80 years old: 25-years’ experience. Intensive Care Med.

[23] White LE, Hassoun HT (2012). Inflammatory mechanisms of organ crosstalk during ischemic acute kidney injury. Int J Nephrol.

[24] Wiedermann CJ, Wiedermann W, Joannidis M (2010). Hypoalbuminemia and acute kidney injury: a meta-analysis of observational clinical studies. Intensive Care Med.

[25] Perez Valdivieso JR, Bes-Rastrollo M, Monedero P (2008). Impact of prealbumin levels on mortality in patients with acute kidney injury: an observational cohort study. J Renal Nutr.

[26] Beier K, Eppanapally S, Bazick HS (2011). Elevation of blood urea nitrogen is predictive of long-term mortality in critically ill patients independent of “normal” creatinine. Crit Care Med.

[27] Liu KD, Himmelfarb J, Paganini E (2006). Timing of initiation of dialysis in critically ill patients with acute kidney injury. Clin J Am Soc Nephrol.

[28] Safavi M, Honarmand A (2007). Admission hypomagnesemia–impact on mortality or morbidity in critically ill patients. Middle East J Anaesthesiol.

[29] Zafar MS, Wani J, Karim R (2014). Significance of serum magnesium levels in critically ill-patients. Int J Appl Basic Med Res.

[30] Limaye CS, Londhey VA, Nadkart MY (2011). Hypomagnesemia in critically ill medical patients. J Assoc Physicians India.

[31] Alves SC, Tomasi CD, Constantino L (2013). Hypomagnesemia as a risk factor for the non-recovery of the renal function in critically ill patients with acute kidney injury. Nephrol Dialysis Transplant.

